# Effectiveness of multidisciplinary rehabilitation on functional recovery in post-COVID-19 patients: a multicentric study across Ecuadorian healthcare centers

**DOI:** 10.3389/fmed.2026.1711031

**Published:** 2026-02-13

**Authors:** Paola Yepez, Juan D. Martinez-Lemus, Alejandra Mafla-Viscarra, Fernando Ortega Pérez, David Sebastián Peña Campos, Jorge Elías Rodríguez Arias, Evelyn Caballero Caballero, Mirely Tobar, Killen H. Briones-Zamora, Killen H. Briones-Claudett, Michelle Grunauer

**Affiliations:** 1 School of Medicine, Universidad San Francisco de Quito, Quito, Ecuador; 2 RISE (Reaching Impact, Saturation and Epidemic Control), Quito, Ecuador; 3 University of Texas Health Science Center, McGovern Medical School, TX, United States; 4 Universidad de Especialidades Espiritu Santo, Samborondon, Ecuador; 5 Universidad Internacional del Ecuador. UIDE, Quito, Ecuador

**Keywords:** functional recovery, post-covid condition, program, rehabilitation, SARS-CoV-2

## Abstract

**Background:**

Post-COVID-19 condition (PCC) poses a major challenge for health systems, particularly in low- and middle-income countries, where evidence on the benefits of multidisciplinary rehabilitation remains limited.

**Objective:**

To evaluate changes in functional and perceived health over 12 weeks of multidisciplinary rehabilitation and to determine whether recovery differed between patients with and without PCC.

**Methods:**

We conducted a multicentric, longitudinal cohort study of adults with confirmed COVID-19 enrolled in a multidisciplinary rehabilitation program. PCFS and AVS were evaluated at baseline and at 4-week intervals through Week 12. Patients were classified by PCC status at enrollment. Group comparisons, longitudinal analyses, and logistic regression models (unadjusted and adjusted) were performed to assess changes over time and factors associated with reduced functional improvement. Analyses were conducted using SPSS V.25 and GraphPad Prism V.10.

**Results:**

A total of 477 patients were enrolled; 354 (74.2%) met PCC criteria at baseline (enrollment), and 123 (25.8%) were classified as non-PCC. Follow-up completion for PCFS was 351 patients at Week 4 (73.6%), 330 at Week 8 (69.2%), and 250 at Week 12 (52.4%). At baseline, PCC patients were younger (*p* = 0.030), had more comorbidities (*p* < 0.001), and differed in education (*p* = 0.007), occupation (*p* = 0.012), and initial provider type (*p* = 0.004), while sex did not differ (*p* = 0.299). Over 12 weeks, PCFS decreased by 0.48 points (95% CI –0.65 to −0.31, *p* < 0.0001) and AVS increased by 0.68 points (95% CI 0.56–0.80, *p* < 0.0001). In adjusted models, baseline PCC was strongly associated with lower odds of PCFS improvement (aOR 0.27, 95% CI 0.13–0.56, *p* < 0.001). AVS improvement did not differ by baseline PCC status (*p* = 0.062).

**Conclusion:**

Multidisciplinary rehabilitation improved both functional status and perceived health; however, patients with baseline PCC showed less functional recovery. These findings underscore the importance of early identification of PCC status at enrollment and emphasize the need for targeted rehabilitation.

## 1 Introduction

The COVID-19 pandemic has triggered an unprecedented global health crisis, exerting significant strain on healthcare systems, economies, and societies worldwide (1). While early response efforts prioritized reducing acute mortality, attention has gradually shifted toward understanding and addressing the long-term effects of the disease (2). One of the most concerning post-acute complications is post-COVID-19 condition (PCC), commonly referred to as “Long COVID,” which has been identified as a substantial cause of morbidity (3). The disease prevalence appears to be influenced by factors such as prior hospitalization and vaccination status (4). In response to this growing burden, rehabilitation programs for PCC have gained importance, not only in improving patient outcomes but also in advancing therapeutic strategies for chronic respiratory conditions (5). The development and refinement of these rehabilitation approaches contribute to optimization of respiratory therapy techniques, with broader applications in the management of other chronic pulmonary diseases, including chronic obstructive pulmonary disease and interstitial lung diseases (6).

PCC is characterized by a wide spectrum of persistent symptoms, including severe fatigue, dyspnea, exercise intolerance, cognitive impairment, psychiatric disorders, and chronic pain (7). The resulting functional decline and diminished quality of life present a significant challenge in post-pandemic healthcare system (8). Given this heterogeneity, pulmonary and physical rehabilitation has been proposed as a key therapeutic strategy to restore functional capacity, alleviate symptoms, and enhance patient independence (9). Although rehabilitation programs have demonstrated efficacy in other respiratory conditions, their specific role and effectiveness in PCC recovery remain to be fully elucidated (10). PCC is clinically defined by the persistence of symptoms for ≥12 weeks after infection, with associated functional impact, and no alternative diagnosis, rather than by the absence of symptoms among those who do not meet the case definition (11).

Understanding the variability in patient responses to rehabilitation is crucial for optimizing care strategies (12). Factors such as demographic characteristics, pre-existing comorbidities, severity of initial infection, predominant symptom clusters, and timing of rehabilitation initiation may all influence outcomes (13). Identifying predictors of favorable responses will enable the refinement of referral criteria and the more efficient allocation of healthcare resources.

Ecuador has experienced a substantial burden from COVID-19, and observational evidence from Ecuadorian cohorts indicates that PCCs are common and clinically relevant in this setting (14). While much attention has been focused on the acute phase of the disease, the long-term sequelae are now emerging as a critical healthcare challenge. Many individuals experience fluctuating symptoms over prolonged periods, significantly impairing daily life and productivity (15). Evaluating the impact of rehabilitation interventions in this population is imperative (16).

This multicentric, prospective longitudinal cohort study assesses the functional impact of a structured multidisciplinary rehabilitation program delivered across 14 healthcare centers in Ecuador between January and June 2023. The changes in functional status and perceived health were evaluated over 12 weeks, and the rehabilitation responses between individuals with and without PCC were compared, all of whom received the same rehabilitation framework. Multivariable regression analyses enabled the identification of demographic, clinical, and healthcare system factors associated with rehabilitation outcomes, and the independent contribution of baseline PCC status to functional recovery was evaluated. By characterizing differential trajectories and determinants of improvement, this study aims to inform the design of evidence-based rehabilitation strategies and guide public health planning for the growing burden of PCC.

## 2 Methods

### 2.1 Study design, setting, and participants

This prospective longitudinal cohort study was conducted between January and June 2023 across 14 institutions in five provinces of Ecuador (Pichincha, Guayas, Azuay, Manabí, and Tungurahua). Adults with confirmed prior SARS-CoV-2 infection were recruited during standard-of-care inpatient and outpatient visits. Enrollment was simplified by providers from general medicine, family medicine, psychology, nutrition, occupational therapy, and respiratory therapy. Inclusion criteria included age 18–75 years, a confirmed SARS-CoV-2 diagnosis at least 12 weeks before enrollment, and the ability to tolerate at least 30 min of physical therapy per day. Exclusion criteria included a positive COVID-19 test within the prior 12 weeks, active infectious diseases, other acute illnesses, or hemodynamic instability, including increasing oxygen requirements of >5 L/min, chronic mechanical ventilation, uncontrolled arrhythmias, active cardiac ischemia, or ongoing deep vein thrombosis or pulmonary embolism. Ethical approval was granted by the San Francisco de Quito University Ethics Committee (IRB No. 2022-068 M). The database was anonymized, and all participants provided written informed consent. No compensation was offered.

### 2.2 Definition of post-COVID-19 condition (PCC)

PCC status was determined once at enrollment (baseline) and was not reassigned during follow-up. PCC was defined according to established clinical criteria as symptoms persisting for ≥12 weeks after confirmed SARS-CoV-2 infection, associated with functional impact, and not attributable to an alternative diagnosis (11). Participants who did not meet the PCC case definition at baseline were classified as non-PCC (i.e., did not fulfill one or more of: symptom persistence ≥12 weeks, functional impact, and no alternative diagnosis). Non-PCC does not imply absence of symptoms; it indicates that the PCC diagnostic threshold was not met at enrollment.

### 2.3 Assessments and rehabilitation protocol

At enrollment, the enrolling provider administered a standardized questionnaire capturing demographics, social determinants of health (SDoH), symptom presentation, comorbidities, SARS-CoV-2 vaccination status, and baseline post-COVID-19 functional status scale (PCFS) (17), as well as a single 5-point Likert patient-reported perceived health improvement item (AVS; “I feel that my current health has improved since I started rehabilitation”). Participants received multidisciplinary rehabilitation within a structured post-COVID framework implemented across all participating sites. Core components were standardized across centers, with delivery adapted to local resources and individualized to patient needs. Therapy was provided three times per week for 8 weeks (Supplementary file 1). Follow-up visits at Weeks 4, 8, and 12 were conducted by the same provider and included repeat PCFS and AVS assessments. Symptom trajectories were documented using the PCC symptom module among participants classified as PCC at baseline; non-PCC participants were not included in symptom-level analyses.

### 2.4 Outcomes

The primary outcomes were changes over time in PCFS and AVS among participants with and without baseline PCC. PCFS (0–4) assesses limitations in usual activities: Grade 0 indicates no limitations, Grade 1 negligible limitations, Grade 2 occasional activity reduction without assistance, Grade 3 inability to perform all usual activities while maintaining self-care, and Grade 4 severe limitations requiring assistance. For logistic regression, a favorable PCFS outcome was defined as Grades 0–1 and unfavorable as ≥2. AVS assessed perceived health improvement using the single 5-point Likert item; a favorable AVS outcome was defined as “Strongly agree,” and unfavorable as ≤4 (Supplementary file 1). Exploratory outcomes included associations between baseline PCC status and Week 12 outcomes using logistic regression models (both unadjusted and adjusted) for controlling age, sex, comorbidities, and SDoH (education, occupation, clinical setting, and initial provider). All regression models used baseline PCC classification as the exposure variable. For logistic regression, “improvement” was operationalized as achieving a favorable outcome at Week 12 (PCFS Grades 0–1; AVS “Strongly agree”).

### 2.5 Sample size

A formal *a priori* sample size calculation was not performed. Sample size was feasibility-based, reflecting adult attendance in participating rehabilitation programs during 2020–2021 and the lack of prior effect-size estimates for functional or perceived health outcomes in similar populations. All eligible participants within the study period were consecutively enrolled.

### 2.6 Statistical analysis

Descriptive statistics were used to summarize baseline characteristics and follow-up completion. Group comparisons between participants with and without PCC employed chi-squared or Fisher’s exact tests for categorical variables and *t*-tests or nonparametric equivalents for continuous variables, as appropriate. Longitudinal changes in PCFS and AVS were analyzed using a mixed-effects repeated-measures model (REML), with time as the within-subject factor, accounting for within-individual correlations and missing follow-up data. When the overall time effect was significant, pairwise comparisons versus baseline at Weeks 4, 8, and 12 were performed using Dunnett’s multiple comparisons test. Associations between baseline PCC status and Week 12 outcomes were assessed using unadjusted and adjusted logistic regression models; for these models, “improvement” was operationalized as achieving a favorable outcome at Week 12 (PCFS Grades 0–1; AVS “Strongly agree”). Results are reported as odds ratio (OR) and adjusted odds ratio (aOR) with 95% confidence intervals (CI). A two-sided *p*-value <0.05 was considered statistically significant. Sensitivity analyses repeated logistic regression models within subgroups defined by the number of completed visits (2, 3, or 4) to assess potential attrition bias. Analyses were conducted using SPSS v25 and GraphPad Prism v10.

## 3 Results

### 3.1 Recruitment, follow-up, and baseline characteristics

A total of 477 patients were enrolled. Follow-up decreased at each assessment point, with 351 patients completing Week 4 (73.6%), 330 completing Week 8 (69.2%), and 250 (52.4%) completing Week 12 evaluations. Follow-up completion did not differ significantly between PCC and non-PCC groups at any timepoint: Week 4 (71.5% vs. 74.3%, *p* = 0.551), Week 8 (69.1% vs. 69.2%, *p* = 0.983), and Week 12 (184/354 [52.0%] vs. 66/123 [53.7%], *p* = 0.748). Additional baseline demographic and clinical characteristics are summarized in Table 1.

**Table 1:** Baseline demographic, clinical, and social determinants of health characteristics of the study population, stratified by PCC status (*n* = 477).

Variable	All	non-PCC	PCC	*p*-value
Age				0.030
18–19	4.0 (0.8%)	2.0 (1.6%)	2.0 (0.6%)	
20–29	49.0 (10.3%)	21.0 (17.1%)	28.0 (7.9%)	
30–39	108.0 (22.6%)	29.0 (23.6%)	79.0 (22.3%)	
40–49	91.0 (19.1%)	19.0 (15.4%)	72.0 (20.3%)	
50–59	105.0 (22.0%)	28.0 (22.8%)	77.0 (21.8%)	
60–69	74.0 (15.5%)	18.0 (14.6%)	56.0 (15.8%)	
≥70	46.0 (9.6%)	6.0 (4.9%)	40.0 (11.3%)	
Female sex	313.0 (65.6%)	76.0 (61.8%)	237.0 (66.9%)	0.299
Education				0.007
None	13.0 (2.7%)	3.0 (2.4%)	10.0 (2.8%)	
Elementary	101.0 (21.2%)	31.0 (25.2%)	70.0 (19.8%)	
Secondary	189.0 (39.6%)	56.0 (45.5%)	133.0 (37.6%)	
Higher education	172.0 (36.1%)	31.0 (25.2%)	141.0 (39.8%)	
Postgraduate	2.0 (0.4%)	2.0 (1.6%)	0.0 (0.0%)	
Occupation				0.012
None	27.0 (5.7%)	8.0 (6.5%)	19.0 (5.4%)	
Student	16.0 (3.4%)	8.0 (6.5%)	8.0 (2.3%)	
Artisan	247.0 (51.8%)	73.0 (59.3%)	174.0 (49.2%)	
Professional	186.0 (39.0%)	34.0 (27.6%)	152.0 (42.9%)	
Other	1.0 (0.2%)	0.0 (0.0%)	1.0 (0.3%)	
Clinical setting				0.879
Urban	282.0 (59.1%)	72.0 (58.5%)	210.0 (59.3%)	
Rural	195.0 (40.9%)	51.0 (41.5%)	144.0 (40.7%)	
Type of provider enrolling				0.004
Emergency medicine	1.0 (0.2%)	0.0 (0.0%)	1.0 (0.3%)	
Family medicine	53.0 (11.1%)	7.0 (5.7%)	46.0 (13.0%)	
General medicine	45.0 (9.4%)	5.0 (4.1%)	40.0 (11.3%)	
Nutrition	1.0 (0.2%)	0.0 (0.0%)	1.0 (0.3%)	
Psychology	1.0 (0.2%)	1.0 (0.8%)	0.0 (0.0%)	
Speech therapy	6.0 (1.3%)	0.0 (0.0%)	6.0 (1.7%)	
Physical therapy	355.0 (74.4%)	109.0 (88.6%)	246.0 (69.5%)	
Occupational therapy	7.0 (1.5%)	0.0 (0.0%)	7.0 (2.0%)	
Respiratory therapy	8.0 (1.7%)	1.0 (0.8%)	7.0 (2.0%)	
COVID-19 vaccine	472.0 (99.0%)	120.0 (97.6%)	352.0 (99.4%)	0.0790
COVID-19 vaccine doses				0.014
One	5.0 (1.1%)	2.0 (1.7%)	3.0 (0.9%)	
Two	45.0 (9.5%)	18.0 (15.0%)	27.0 (7.7%)	
Three	149.0 (31.6%)	46.0 (38.3%)	103.0 (29.3%)	
Four	271.0 (57.4%)	53.0 (44.2%)	218.0 (61.9%)	
Five	2.0 (0.4%)	1.0 (0.8%)	1.0 (0.3%)	
Presence of comorbidities	285.0 (59.7%)	41.0 (33.3%)	244.0 (68.9%)	<0.001
Number of comorbidities				<0.001
None	192.0 (40.3%)	82.0 (66.7%)	110.0 (31.1%)	
One	208.0 (43.6%)	27.0 (22.0%)	181.0 (51.1%)	
Two	60.0 (12.6%)	13.0 (10.6%)	47.0 (13.3%)	
Three	16.0 (3.4%)	1.0 (0.8%)	15.0 (4.2%)	
Five	1.0 (0.2%)	0.0 (0.0%)	1.0 (0.3%)	
Baseline functional status (PCFS grades)				<0.001
Grade 0	216.0 (45.3%)	45.0 (36.6%)	171.0 (48.3%)	
Grade 1	120.0 (25.2%)	50.0 (40.7%)	70.0 (19.8%)	
Grade 2	39.0 (8.2%)	5.0 (4.1%)	34.0 (9.6%)	
Grade 3	45.0 (9.4%)	7.0 (5.7%)	38.0 (10.7%)	
Grade 4	57.0 (11.9%)	16.0 (13.0%)	41.0 (11.6%)	
Baseline perceived health (AVS categories)				0.597
Strongly disagree	14.0 (3.4%)	2.0 (2.0%)	12.0 (3.9%)	
Disagree	1.0 (0.2%)	0.0 (0.0%)	1.0 (0.3%)	
Neutral	58.0 (14.2%)	11.0 (10.9%)	47.0 (15.3%)	
Agree	192.0 (47.1%)	52.0 (51.5%)	140.0 (45.6%)	
Strongly agree	143.0 (35.0%)	36.0 (35.6%)	107.0 (34.9%)	

Values are *n* (%). Percentages are calculated using the number of participants with available data for each variable. Vaccine-dose percentages are calculated among vaccinated participants (All *n* = 472; non-PCC *n* = 120; PCC *n* = 352). AVS percentages are calculated among participants who completed baseline AVS (All *n* = 408; non-PCC *n* = 101; PCC *n* = 307). *p*-values from *χ*^2^ or Fisher’s exact tests. PCC, post-COVID-19 condition; PCFS, post-COVID-19 Functional Status Scale; AVS, 5-point Likert patient-reported perceived health improvement item (“I feel that my current health has improved since I started rehabilitation”).

### 3.2 PCC classification and baseline group differences

Of the 477 enrolled patients, 354 (74.2%) met criteria for PCC at baseline (enrollment), and 123 (25.8%) were classified as non-PCC. Sex distribution did not differ between participants with and without PCC (*p* = 0.299). In contrast, age differed significantly, with PCC participants being younger at enrollment (*p* = 0.030). Individuals with PCC also had a higher comorbidity burden (*p* < 0.001). Several SDoH varied by PCC status, including education level (*p =* 0.007), occupation (*p =* 0.012), and initial provider type (*p =* 0.004), while no differences were observed in the clinical setting of care.

### 3.3 Post-COVID-19 condition symptom profile

**Figure 1:**
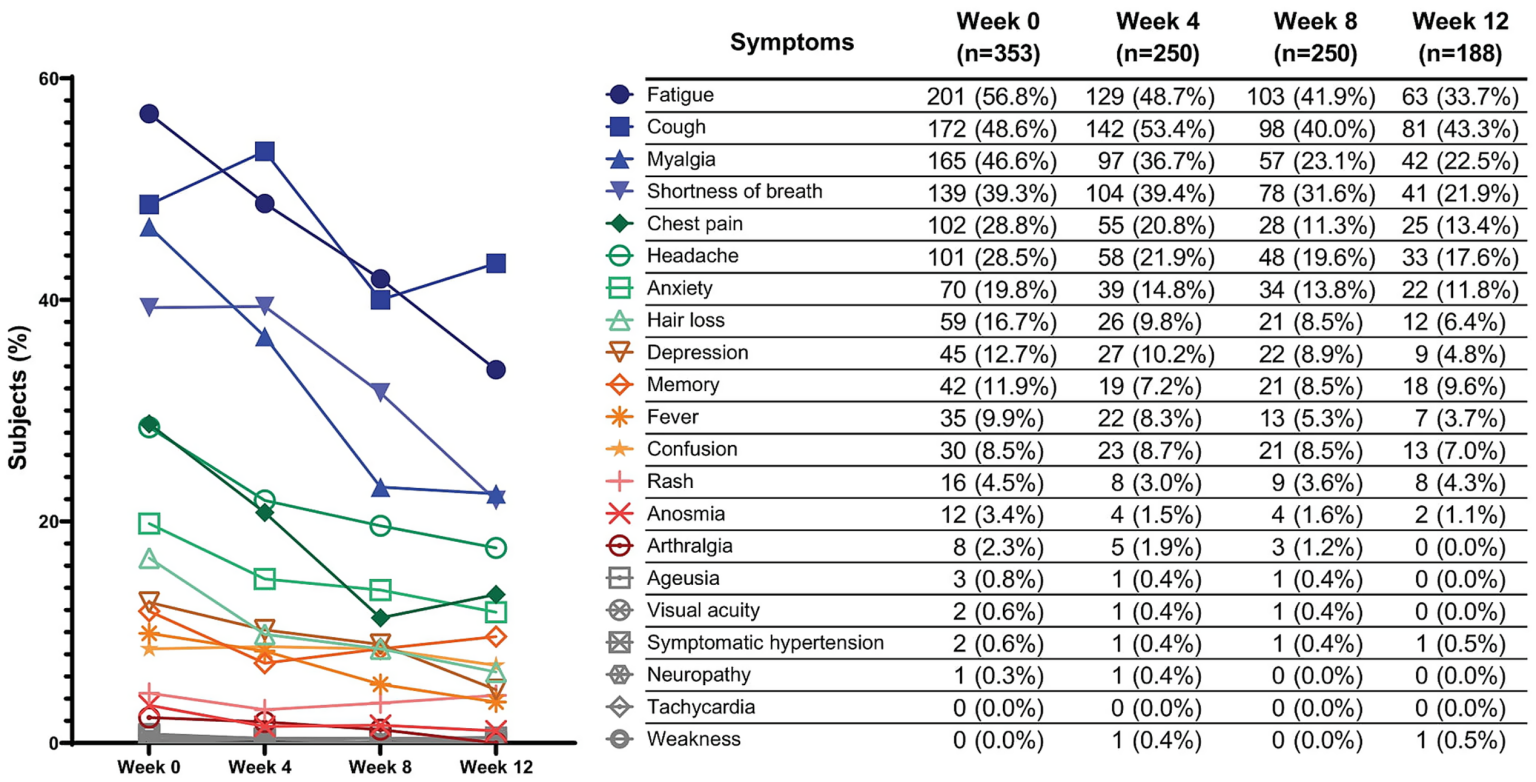
Symptom prevalence and trajectory among participants classified as PCC at baseline (PCC subgroup only). Symptom data were collected within the PCC symptom module and are reported as the proportion of participants with available symptom data at each time point. Baseline symptom data were available for 353/354 PCC participants (week 0, *n* = 353). Denominators at follow-up were week 4 (*n* = 250), week 8 (*n* = 250), and week 12 (*n* = 188) due to loss to follow-up/missing symptom forms. Non-PCC participants were not included in symptom-level analyses, and symptom reporting did not affect PCC classification, which was defined at baseline.

Among PCC participants with available symptom data (353/354), the most frequent baseline symptoms were fatigue (56.8%), cough (48.6%), myalgia (46.6%), and shortness of breath (39.3%). Overall symptom burden decreased over the 12-week rehabilitation period (Figure 1). By Week 12, fatigue had decreased to 33.7%, myalgia to 22.5%, and dyspnea to 21.9%. Psychological symptoms (anxiety and depression) and other less common complaints generally declined over time. Symptom trajectories in Figure 1 correspond to the PCC subgroup only; non-PCC participants were not included in symptom-level analyses.

### 3.4 Changes in PCFS over time and association with outcomes

**Figure 2:**
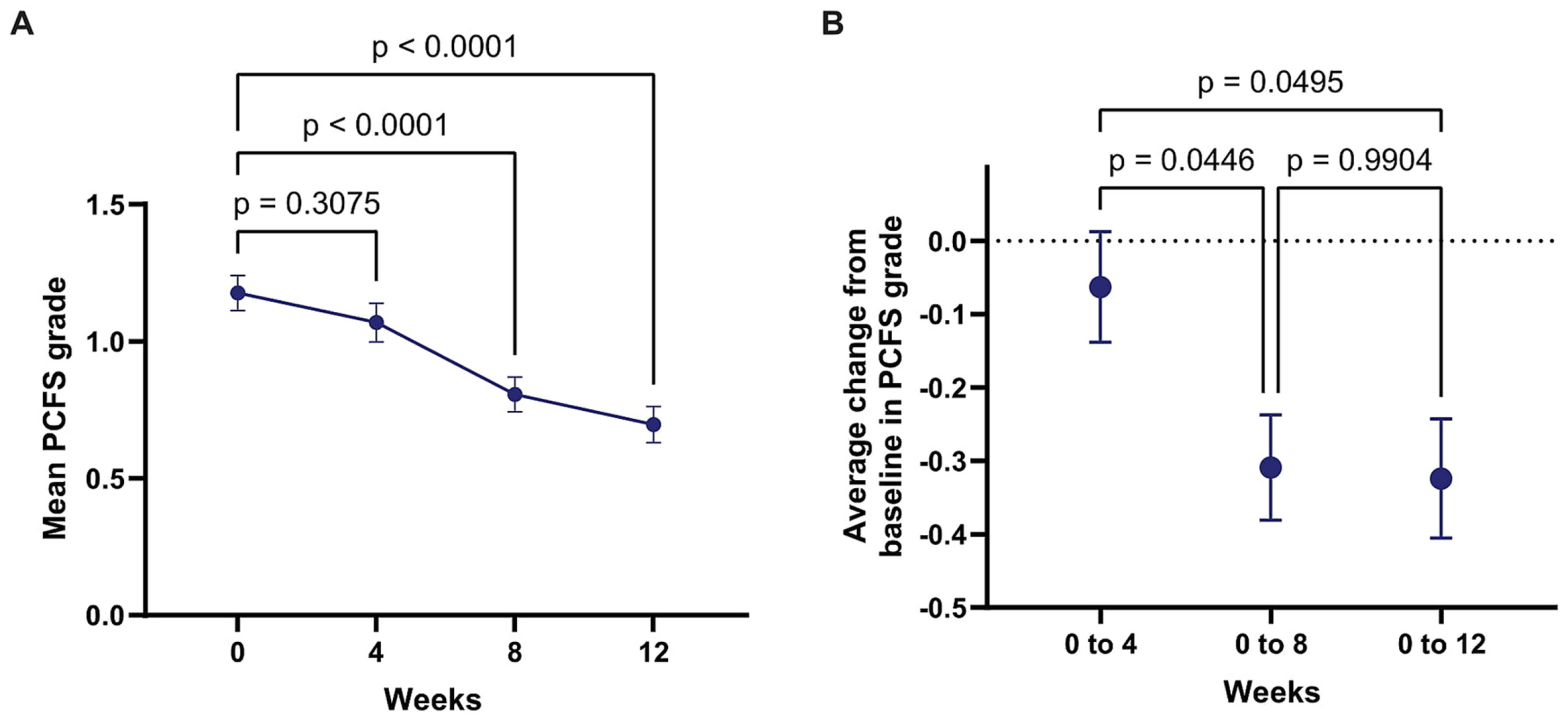
Change in post-COVID-19 functional status (PCFS) over 12 weeks. **(A)** Mean PCFS grade at each timepoint. *p*-values represent pairwise comparisons versus baseline using Dunnett’s multiple comparisons test (week 4: *p* = 0.3075; week 8: *p* < 0.0001; week 12: *p* < 0.0001). **(B)** Mean change from baseline in PCFS grade. *p*-values test whether the mean change differs significantly from zero at each interval (0–4 weeks: *p* = 0.9904; 0–8 weeks: *p* = 0.0446; 0–12 weeks: *p* = 0.0495). Overall effect of time was significant in the mixed-effects repeated-measures model (*p* < 0.0001).

All enrolled patients completed the baseline PCFS assessment. Follow-up patterns varied: 107 patients (22.4%) completed baseline only, 33 (6.9%) completed baseline plus one follow-up (Weeks 0–4), 113 (23.7%) completed baseline plus two follow-ups (Weeks 0–8), and 224 (46.9%) completed baseline plus all three follow-ups (Weeks 0–12). PCFS scores improved significantly over the 12-week rehabilitation period (Figure 2). The mixed-effects repeated-measures model demonstrated a significant effect of time (*p* < 0.0001), with mean PCFS decreasing by 0.11 points at Week 4 (*p* = 0.3075), 0.37 points at Week 8 (*p* < 0.0001), and 0.48 points at Week 12 (95% CI –0.65 to −0.31, *p* < 0.0001). In unadjusted logistic regression, patients with baseline PCC had substantially lower odds of PCFS improvement (OR 0.27, 95% CI 0.14–0.55, *p* < 0.001). In the adjusted model, baseline PCC remained strongly associated with reduced odds of improvement (aOR 0.27, 95% CI 0.13–0.56, *p* < 0.001). Among covariates, older age was independently associated with greater likelihood of improvement (aOR 1.35, 95% CI 1.12–1.62, *p* = 0.002), while initial provider type was also significant, with patients initially enrolled through non-rehabilitation services showing lower odds of PCFS improvement (aOR 0.71, 95% CI 0.57–0.89, *p* = 0.002; Supplementary file 2).

### 3.5 Changes in AVS over time and association with outcomes

A total of 408 (85.5%) enrolled patients completed the baseline AVS assessment. Follow-up patterns varied: 104 patients (25.5%) completed baseline only, 69 (16.9%) completed baseline plus one follow-up (Weeks 0–4), 138 (33.8%) completed baseline plus two follow-ups (Weeks 0–8), and 97 (23.8%) completed baseline plus all three follow-ups (Weeks 0–12). AVS scores improved significantly over the 12-week rehabilitation period, with a significant effect of time (*p* < 0.0001). Mean AVS increased by 0.18 points at Week 4 (*p* = 0.042), 0.33 points at Week 8 (*p* < 0.001), and reached a 0.68-point improvement at Week 12 (95% CI 0.56 to 0.80, *p* < 0.0001). In unadjusted logistic regression, AVS improvement was not associated with baseline PCC status (OR 0.65, 95% CI 0.39–1.11, *p* = 0.113). The association remained non-significant after adjustment (aOR 0.58, 95% CI 0.33–1.03, *p* = 0.062). Significant covariates included age, with older age associated with higher odds of perceived health improvement (aOR 1.45, 95% CI 1.18–1.78, *p* < 0.001), and initial provider type, where patients enrolled through general care services had lower odds of AVS improvement compared with those enrolled via rehabilitation-specialty providers (aOR 0.72, 95% CI 0.57–0.92, *p =* 0.007; Supplementary file 2).

### 3.6 Sensitivity analyses

To determine whether follow-up completeness influenced the association between PCC and outcomes, logistic regression models were repeated within subgroups of participants with 2, 3, and 4 completed visits. For PCFS, the 2-visit subgroup had an OR of 4.37 (95% CI 0.16–122.30, *p =* 0.386), the 3-visit subgroup had an OR of 1.92 (95% CI 0.65–5.69, *p =* 0.241), and the 4-visit subgroup had a significant OR of 0.025 (95% CI 0.003–0.191, *p* < 0.001). For AVS, no subgroup showed a significant association, with ORs of 1.36 (*p =* 0.701) for the 2-visit subgroup, 0.58 (*p =* 0.244) for the 3-visit subgroup, and 1.07 (*p =* 0.923) for the 4-visit subgroup. Full subgroup models are presented in Supplementary file 3.

## 4 Discussion

This multicenter cohort study found that a structured post-COVID-19 multidisciplinary rehabilitation program was associated with improved functional status over 12 weeks, with substantial differences in recovery according to baseline PCC status. Individuals with baseline PCC had markedly lower odds of achieving a favorable PCFS outcome compared with non-PCC participants. In unadjusted analyses, baseline PCC was associated with a 73% reduction in the odds of functional improvement (OR 0.27, 95% CI 0.14–0.55), and this association remained unchanged after adjustment for age, sex, comorbidity burden, education, and provider type (aOR 0.27, 95% CI 0.13–0.56). Together, these findings indicate that baseline PCC status was independently associated with reduced functional recovery during follow-up.

Long COVID has been reported in a substantial proportion of SARS-CoV-2 survivors, with estimates ranging from approximately 10 to 30%, depending on the population, follow-up duration, and case definition (13, 15). Large cohort studies have identified female sex, middle age, higher body mass index, and greater acute disease severity as consistent predictors of persistent symptoms (13, 18). In addition, longitudinal analyses highlight the heterogeneity and fluctuating nature of symptom trajectories, with multisystem involvement and prolonged functional impact in a subset of patients (13, 15, 16).

Although no sex-based differences were observed (*p =* 0.299), participants with baseline PCC were younger at enrollment (*p =* 0.030) yet had a higher comorbidity burden (*p* < 0.001), indicating a more complex clinical profile despite their younger age (18). Educational attainment differed between groups, with higher education more frequent among participants with baseline PCC, and occupation also differed, with artisanal occupations more common in the non-PCC group and professional occupations more common in the PCC group. These baseline differences may reflect socioeconomic or healthcare-access patterns that influence referral pathways and clinical presentation (19, 20).

In addition to baseline PCC status, older age and initial provider type were independently associated with PCFS improvement. Older age was associated with higher odds of improvement (aOR 1.35, 95% CI 1.12–1.62; *p* = 0.002). Patients initially enrolled through non-rehabilitation services also had lower odds of improvement (aOR 0.71, 95% CI 0.57–0.89; *p* = 0.002), consistent with reports of post-COVID rehabilitation cohorts and evidence supporting structured rehabilitation among adults with long COVID (21, 22). Evidence from other post-acute respiratory cohorts has revealed persistent functional limitations after severe COVID-19 compared with non-COVID respiratory illness (23).

Symptom burden at baseline was substantial within the PCC subgroup, with fatigue, cough, myalgia, and dyspnea being the most frequent complaints. These symptoms declined during the 12-week follow-up; however, because symptom trajectories were analyzed within the PCC subgroup only, these changes should be interpreted as within-group trends among participants classified as PCC at baseline (24). These trajectories should also be interpreted in light of attrition over time and the absence of a non-rehabilitation control group, which precludes definitive causal inference.

Although functional status improved overall according to PCFS, multivariable models indicated that participants with baseline PCC had lower odds of achieving a favorable PCFS outcome at Week 12 (aOR 0.27, 95% CI 0.13–0.56). This may partly reflect the more favorable baseline functional status observed in the non-PCC group and greater residual limitations among individuals with baseline PCC. In contrast, AVS improvement did not differ significantly by baseline PCC status, suggesting that perceived health gains may progress similarly despite differences in objective functional recovery.

Attrition was substantial over the 12-week period, with nearly half of participants not completing the final assessment. Sensitivity analyses suggested that the association between baseline PCC and reduced PCFS improvement was statistically significant only among participants completing all follow-up visits, likely reflecting greater statistical power and more precise estimates in that subgroup. In contrast, baseline PCC was not associated with AVS improvement in any subgroup. Barriers to follow-up include persistent symptoms, transportation challenges, competing responsibilities, and the time burden of repeated assessments (25, 26).

However, this study has several limitations. The observational design precludes causal inference, the sample size was feasibility-based, and attrition may introduce bias despite sensitivity analyses (26). Rehabilitation delivery was structured but not fully standardized across centers, potentially introducing variability in exposure. Outcomes relied on patient-reported measures (PCFS and AVS), which capture different dimensions of recovery and may differ in sensitivity to change (17). Residual confounding cannot be excluded, and generalizability may be limited to similar rehabilitation-attending populations.

In conclusion, this multicenter cohort provides evidence that multidisciplinary rehabilitation was associated with enhanced functional outcomes after COVID-19, although individuals with baseline PCC experienced less functional improvement. Baseline PCC status remained independently associated with lower odds of PCFS improvement, while older age and initial provider type were also associated with recovery. These findings support the need for future randomized controlled trials comparing standardized rehabilitation with usual care to determine therapeutic efficacy and to inform equitable, patient-centered post-COVID-19 care strategies.

## Data Availability

The raw data supporting the conclusions of this article will be made available by the authors, without undue reservation.
