# Supplementary material for: Effectiveness of multidisciplinary rehabilitation on functional recovery in post-COVID-19 patients: a multicentric study across Ecuadorian healthcare centers
Source: Front Med (Lausanne). 2026 Feb 13;13:1711031. doi: 10.3389/fmed.2026.1711031 (PMC12959126; doi:10.3389/fmed.2026.1711031)

**1. Respiratory Therapy**

Goal: improve ventilation, respiratory muscle strength, and dyspnea control

Exercises included:

- Diaphragmatic breathing in 4 positions: supine, prone, sitting, and standing.
- “Yawn-to-smile” breathing with upper-body mobility.
- Humming exhalation (“hum”) to increase nitric oxide and improve alveolar ventilation.
- Inspiratory Muscle Training (IMT):
  - 3 sets of 5 minutes (1 min work / 1 min rest).
  - Initial resistance at 40–50% of PIMax (or at tolerated level if PIMax is not measured).
  - Resistance increased by ~5% every two weeks.

**2. Aerobic Exercise**

Modalities: walking, brisk walking, stationary cycling, slow jogging, or marching in place.

Progression:

- Weeks 1–4: low intensity, 20–30 minutes/session.
- Weeks 5–12: gradual progression to moderate intensity as tolerated.
- Training methods:
- Interval method: 1 min activity / 1 min rest at the beginning.
- Continuous method: progression from 3–15 minutes continuous work with 3-minute rest intervals.

**3. Resistance Training**

Goal: rebuild strength and muscle mass.

Exercises included:

- Mini-squats with controlled breathing.
- Heel raises (progressing from supported to unsupported, then with arms elevated).
- Sit-to-stand with stable gaze.
- Arm curls with light weights (1–2 lb).
- Overhead arm raises with light weights.
- Dosage: 1–3 sets, 3–4 times/week (included within the 60-min sessions).
- Progression: increased load or repetitions every 2 weeks.

**4. Balance and Mobility**

Goal: improve functional mobility, gait confidence, and tolerance to daily activities.

Marching in place with high knees.

Modified jumping jacks (low-impact).

Forward lunges with postural control.

**6. Psychological Support**

- Relaxation breathing and mindfulness-based techniques.
- Short guided meditation (10–15 minutes).
- Identification of anxiety triggers associated with exertion.
- Weekly goal-setting and adherence review.

Although the rehabilitation framework was based on these standardized components, each health center and hospital adapted the protocol according to their available resources, and the therapy was individualized for each patient. Therefore, not all patients performed the exact same exercises or sequence, but every participant received a structured, directed Post COVID-19 rehabilitation program tailored to their clinical needs. This protocol outlines the unified post COVID-19 rehabilitation program that was already part of routine clinical practice at all participating sites. Every participant in the study regardless of whether they had PCC underwent the same intervention. The program was not allocated through a randomized or controlled trial; rather, its effects were evaluated within a prospective cohort framework.

The multidisciplinary rehabilitation protocol was delivered over twelve weeks, with three supervised sessions per week lasting sixty minutes each. The intervention included aerobic training, resistance exercises, respiratory therapy, balance and mobility work, and education and psychological support. Respiratory therapy focused on improving ventilation, respiratory muscle strength, and dyspnea control. Patients performed diaphragmatic breathing in four positions (supine, prone, sitting, and standing), coordinated breathing movements such as the “yawn-to-smile” technique, and humming exhalations to promote nitric oxide production and alveolar ventilation. Inspiratory muscle training (IMT) was conducted with the device for three to fiveminute sets that alternated one minute of work and one minute of rest; resistance was initially set at 40–50% of the patient’s inspiratory pressure or at a tolerated level when maximal pressures were not measurable, with gradual increases every two weeks. Expiratory muscle strengthening followed similar principles. All respiratory exercises were performed with safety monitoring, particularly to avoid dizziness, excessive desaturation, or worsening dyspnea.

Aerobic training involved modalities such as walking, brisk walking, stationary cycling, slow jogging, or marching in place. During the first four weeks, patients exercised at low intensity for twenty to thirty minutes per session, progressing to moderate intensity as tolerated during weeks five to twelve. Depending on the patient’s condition, sessions used either one-to-one minute activity-rest intervals or a variable continuous method that gradually increased sustained activity from three to fifteen minutes. Throughout all sessions, oxygen saturation, heart rate, and blood pressure were monitored to maintain safe parameters.

Resistance training was incorporated to restore muscle strength and mass, particularly in the lower limbs and trunk. Exercises included mini-squats with controlled breathing, heel raises progressing from supported to unsupported, sit-to-stand repetitions with stable visual focus, and upper extremity strengthening with light hand weights. Patients generally performed one to three sets, with increases in load or repetitions every two weeks according to tolerance. Balance and mobility were also addressed through marching in place with knee elevation, modified low-impact jumping movements, and forward lunges, all aimed at improving stability, gait confidence, and daily functional capacity.

Education and self-management formed an essential part of the intervention. Patients were instructed in pacing strategies, techniques for conserving energy, and the recognition of symptom fluctuations. They were taught how to monitor their oxygen saturation, heart rate, perceived exertion, and warning signs that required stopping activity. Psychological support included guided breathing for relaxation, brief mindfulness-based practices such as the Isha Kriya meditation, and the establishment of weekly goals to improve adherence and reduce anxiety related to exertion.

A structured home program complemented supervised sessions. On non-therapy days, patients practiced ten to fifteen minutes of diaphragmatic breathing and humming exercises, performed a twenty-minute low-intensity walk, and completed one to two strength exercises such as heel raises or sit-to-stand repetitions. They also kept daily records of symptoms, oxygen saturation, fatigue scores, and functional status measures. In every session, clinicians monitored oxygen saturation, heart rate, blood pressure, perceived dyspnea, and symptoms. Sessions were stopped if oxygen saturation dropped below 92% or decreased by three percent or more, or if patients developed chest pain, dizziness, visual changes, severe fatigue, or unacceptable dyspnea. Functional assessments, including the Post-COVID Functional Status Scale, symptom visual analogue scales, adherence tracking, the six-minute walk test, and the thirty-second sit-to-stand test, were performed at baseline and again at weeks 4, 8, and 12.

Although the intervention was grounded in these standardized rehabilitation components, each health center and hospital adapted the protocol according to its resources, and therapy was personalized for each patient. As a result, not all individuals completed the exact same exercises or progression; however, all participants received a structured and directed Post-COVID rehabilitation program tailored to their clinical needs and functional capacity.
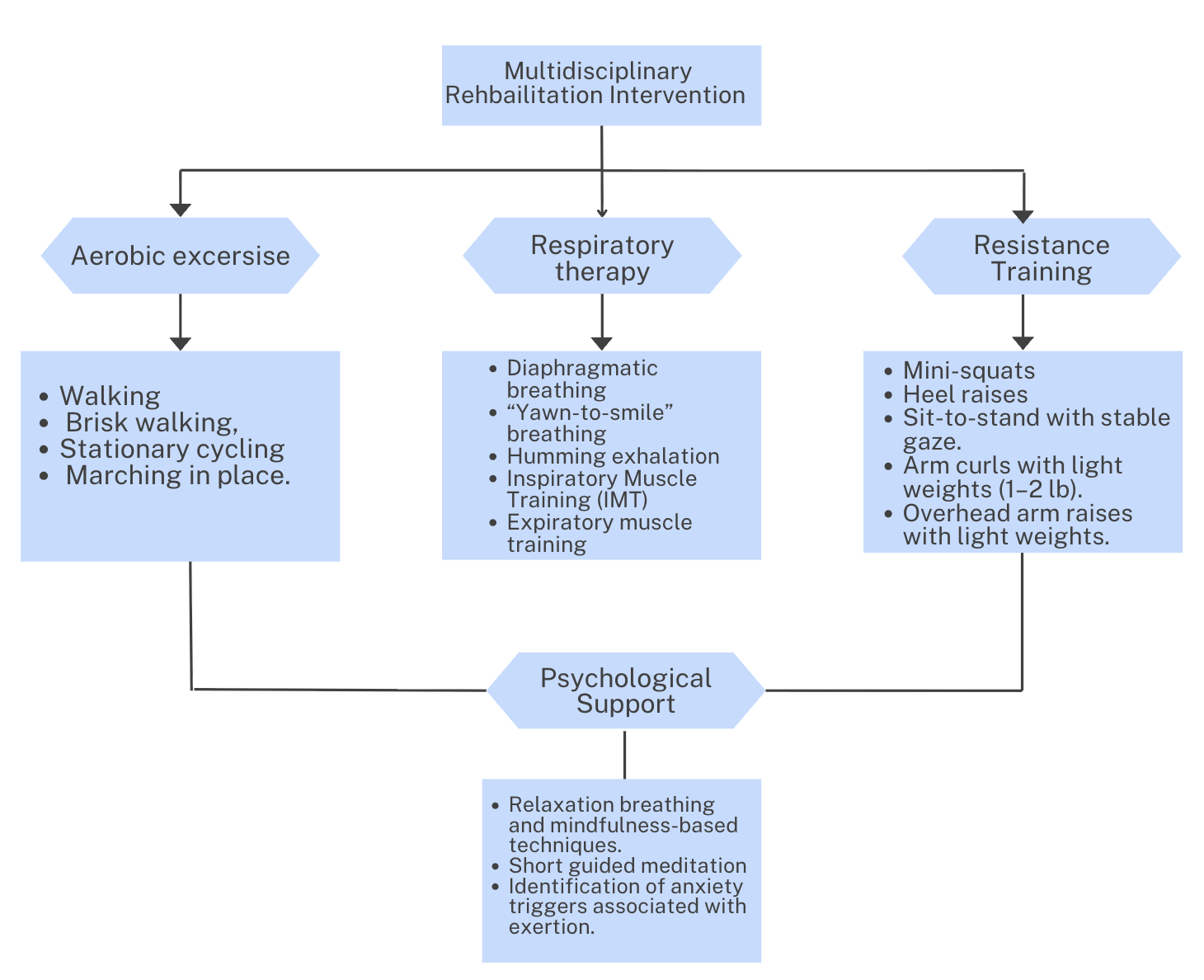


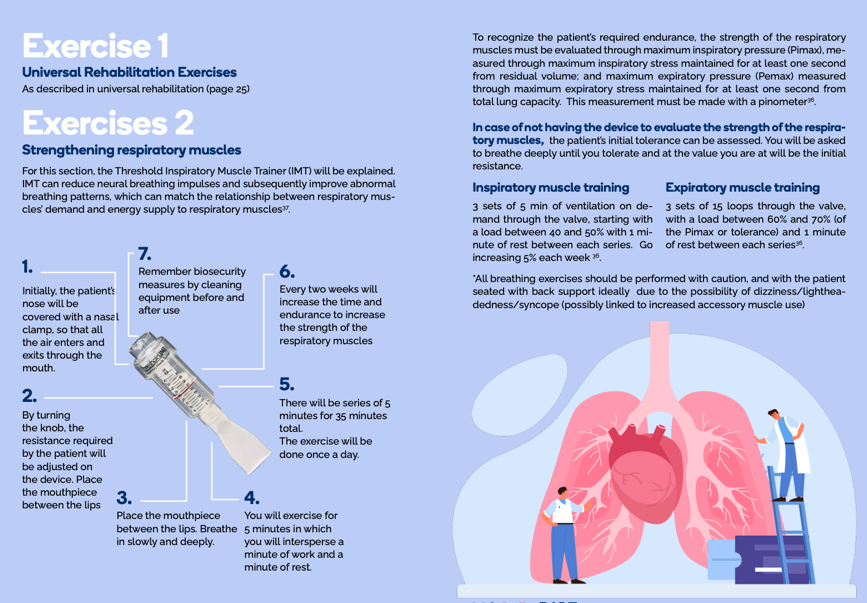

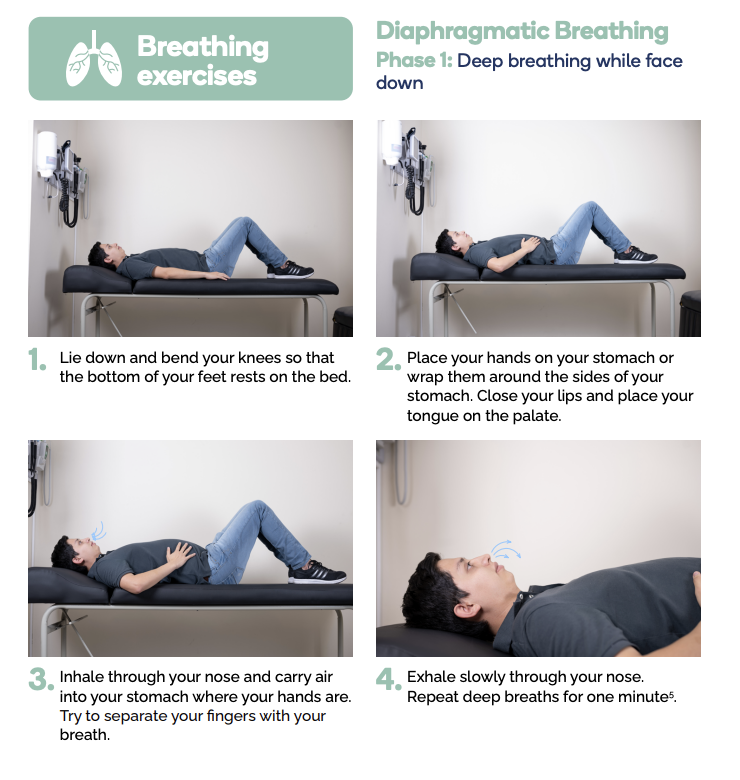


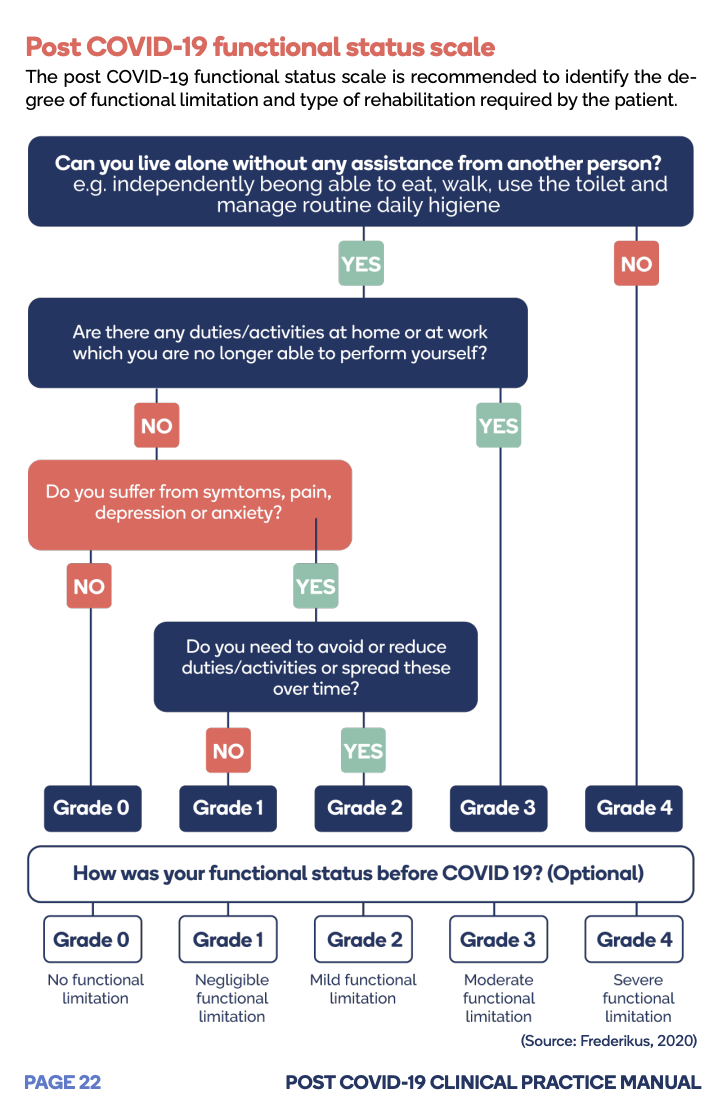

Supplement: Supplementary file 1 [file Supplementary_file_1.docx]
